# Systematical study of multi-walled carbon nanotube nanofluids based disposed transformer oil

**DOI:** 10.1038/s41598-020-77810-8

**Published:** 2020-12-02

**Authors:** Nur Sabrina Suhaimi, Muhamad Faiz Md Din, Mohd Taufiq Ishak, Abdul Rashid Abdul Rahman, Maslina Mohd Ariffin, Nurul ‘Izzati Hashim, Jianli Wang

**Affiliations:** 1 Faculty of Engineering, National Defence University of Malaysia, 57000 Kuala Lumpur, Malaysia; 2 Department of Electrical and Electronic Engineering, University Malaysia Sarawak, 94300 Kota Samarahan, Sarawak Malaysia; 3 Institute for Superconductivity and Electronic Materials, University of Wollongong, Wollongong, NSW 2522 Australia

**Keywords:** Engineering, Electrical and electronic engineering

## Abstract

In this paper, the electrical, dielectric, Raman and small angle X-ray scattering (SAXS) structure behavior of disposed transformer oil in the presence of multi-walled carbon nanotube (MWCNT) were systematically tested to verify their versatility for preparing better alternative transformer oil in future. MWCNT nanofluids are prepared using a two-step method with concentrations ranging from 0.00 to 0.02 g/L. The test results reveal that 0.005 g/L concentration possesses the most optimum performance based on the electrical (AC breakdown and lightning impulse) and dielectric (permittivity, dissipation factor and resistivity) behavior. According to the trend of AC breakdown strength and lightning impulse pattern, there were 212.58% and 40.01% enhancement indicated for 0.005 g/L concentration compared to the disposed transformer oil. The presence of MWCNT also yielding to the decrement of dissipation factor, increased on permittivity and resistivity behavior of disposed transformer oil which reflected to the performance of electrical properties. Furthermore, it is found that these features correlated to the structural properties as systematically verify by Raman and SAXS analysis study.

## Introduction

Significant attention has been paid to the development of insulating oil in transformer, which serves as insulation and coolant medium in transformer. This is due to insulation that is the primary issue that impacts transformer efficiency and reliability. One of the initiatives is the implementation of nanotechnology in insulators with the aim of improving their electrical and thermal behavior. Modern technology has enabled the production of materials with average particle sizes below 100 nm with superior electrical, mechanical, thermal, magnetic and optical properties. Expanding interest in nanomaterials as a future for science and technology constantly attract many researchers, scientists and academician. One of the most commonly studied group of nanomaterials are nanofluid; nanometer-sized particles being dispersed and stable suspended in the base fluid such as water, oils, and alcohols. Nanofluids are often termed as ‘smart fluid’ due to its multifarious and significant application, for example: cooling of electronics, engine cooling, chillers, solar water heating, multichannel heat exchanger and many others^1–4^. Recently, nanofluids have been found exhibit significant enhancement in term of electrical and thermal behavior for transformer oil. As example, TiO_2_ nanofluids have been examined for breakdown strength by Wang et al.^5^, and they presented that the mechanism of nanoparticles have strong electron capture capability and reducing the net density of space charge in transformer oil. Similar natures were presented by Rajeswari et al.^6^ for SiO_2_ nanofluids and Chen et al.^7^ for C60 nanofluids. Most studies have shown that nanofluid could possibly act as a good insulation medium despite its electrical performance. Nonetheless, on the basis of studies, the correct kinds of nanomaterials, the appropriate amount of concentrations, the proper methodology and other considerations are essential in order to provide optimal electrical properties^8^.

Series of studies also have been conducted on dielectric properties of nanofluids, where added surface modified nanoparticles in the transformer oil produced better dissipation factor (DF), DC resistivity and relative permittivity^9^. Although most of researchers claim that nanofluids could contribute to better insulating system and prepared to withstand over-voltages circumstances caused by lightning or switching events, however, information on suspension of nanomaterials with disposed and wasted transformer oil are very limited. Hence, the main contribution of this study is to retreat, recycle and reused disposed transformer oil by dispersing MWCNT with diameter of less than 8 nm to enhance its insulation performance as outlined by IEC standard. Among various nanostructures, MWCNT has unique properties that attracted the interest of researchers for industrial application in term of thermo-physical, electrical, and mechanical characteristics^10^. On the other hand, previous researchers verify that synthetization of MWCNT with new transformer oil is a promising alternative oil for transformer application^11^. The insulation system of MWCNT nanofluid based on disposed mineral oil at varies concentration levels have been studied in this paper. The electrical breakdown voltages (BDV) under AC and standard lightning impulse (LI) under negative polarity were investigated based on IEC 60156^12^ and IEC 60897^13^ test method. The dielectric properties, heat flow Raman and SAXS structure behavior of nanofluid also have been observed respectively.

## Methods

### Nanofluids preparation and measurement

**Figure 1:**
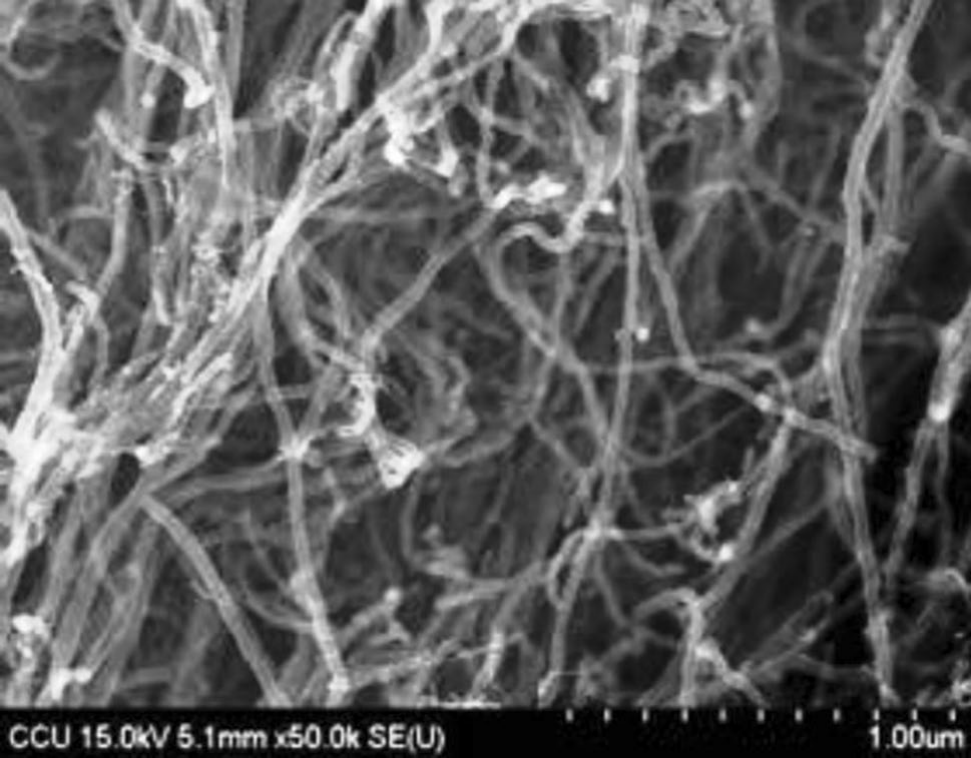
SEM image of Multi-walled carbon nanotube with 5–15 nm diameter size.

MWCNT nanofluids were prepared by synthesizing MWCNT and petroleum-based mineral oil (MO) that has been disposed and wasted by an industry because of its quality that does not meet the IEC standard requirement. The MO used in this study generally contains benzene C_6_H_6_, hexane C_6_H_14_, cyclohexane C_6_H_12_ and etc. The transformer oil has been operated for 10 years in 33/11.5 kV and 16/20MVA rating transformer since 15th October 2009. The purification or reconditioning process is a compulsory requirement for reusing transformer oil in transformer. This is for removing moisture contents that leads to oxidation, dissolved combustible gas and physical contamination contains in the disposed transformer oil. Firstly, the oil was filtered by Nalgene Rapid-Flow disposable filter with 0.2 µm pore size for three repetitive times. After filtering process, the MWCNT with 5–15 nm outer diameter sizes at various concentrations (0.001 g/L, 0.005 g/L, 0.01 g/L, 0.015 g/L and 0.020 g/L) were added into the disposed oil and stirred using magnetic stirrer at room temperature with 520 rpm agitation for 30 min duration. Figure 1 depict the Scanning electron microscope image (SEM) of MWCNT structure. The resulting mixture was subsequently placed in a Q700 sonicator for 120 min with 40% rated power, 20 kHz capability and 700 W power rating. After sonication process, the mixture or known as MWCNT nanofluid is being heated and degassed respectively using a vacuum drying oven at 60 °C and 10Mbar for 24 h^14^.

The AC BDV tests is conducted using a DTA 100C BAUR oil BDV tester along with 400 ml glass test vessel containing two semi-elliptical steel electrodes and at a distance of 2.5 ± 0.05 mm. As for lightning impulse test, the rising method is implemented by injecting one impulse shot per step voltage of 10 kV to the oil samples until breakdown occurs. This experiment is being operated in high voltage laboratory. Permittivity, dissipation factor and resistivity are key parameters for evaluate insulating oil condition in transformer application, which has been carried out ambient temperature until 120 °C to study the aforementioned properties by utilizing *ADTR-2K PLUS* equipment. Furthermore, Raman spectrometer (Renishaw In Via Raman Microscope) is an effective instrument to characterize the detailed bonding structure state of oil samples with different condition. Lastly, Small Angle X-ray Scattering instrument is performed to characterize the uniformity dispersion of MWCNT nanomaterial in disposed transformer oil.

## Results

### Electrical performance

**Figure 2:**
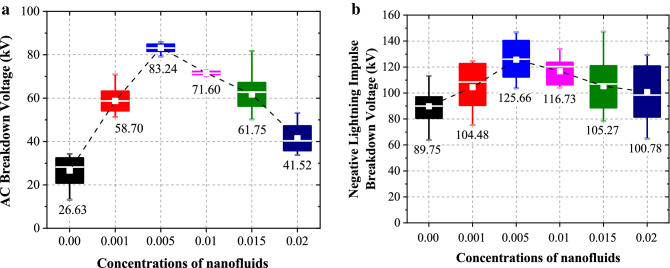
The electrical properties of (**a**) AC breakdown voltage measurements, (**b**) Negative lightning impulse measurements at various concentrations.

Figure 2a depicts the box chart graph of AC BDV of oil samples at different concentrations ranging from 0.00 to 0.02 g/L. It can be seen that there is a clear upward trend and a significant enhancement in disposed MO after dispersed with 0.005 g/L MWCNT as much as 212.58%. Not only that, the standard deviation value is extremely small, which means that the 0.005 g/L MWCNT nanofluid’s BDV are clustered around the mean value (83.24 kV) approximately. Previous authors have explained this circumstance, which explained that nano-sized particles capability to trap electron in the oil and provide better performance of BDV^15^.The mechanism of breakdown in insulation is attributed to the process of hopping electrons in traps and transmitting electrons in a delocalized state. Because of this repeatable trapping and de-trapping process, the rapid electron will be shifted as a slower electron while moving from high electric field region to low region.

Figure 2b shows the results of negative LI breakdown measurements of oil samples, where it can be noticed that the average BDV of 0.005 g/L MWCNT nanofluid shows a significant increment after being sonicated with MWCNT nano-filler as much as 40.01% compared to based oil. This may be due to formation of a significant amount of space charges during the ionization process^7^. Accumulation of space in oil samples is regarded to be one of the major elements that could have a negative impact on the electrical field and influence the efficacy of the breakdown system. In the meantime, as the produced electrons have a higher motion speed, lighter mass, and are capable of travelling through the molecules opposed to positive charges, the electrons eventually began to sweep away from the ionization region. The rapid electrons allow the electric field wave to propagate across the insulation layer, which contributes to a breakdown event. In the meantime, MWCNT consist of high electron adsorption ability known as electro-negativity, which assists in a decrement of number of electrons involved in the development of electron avalanche under electric field. This occurrence disrupts the streamer formation and improves the LI behavior respectively.

### Dielectric properties

**Figure 3:**
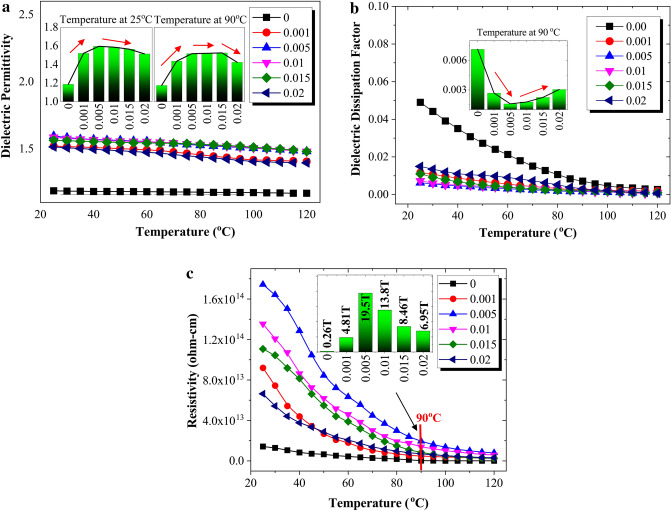
The dielectric properties of oil samples at ascending temperature; (**a**) Dielectric permittivity, (**b**) Dielectric dissipation factor and (**c**) DC resistivity.

The studies of dielectric properties that will be discussed in this paper are dielectric permittivity, dielectric dissipation factor (loss angle) and DC resistivity as shown in Fig. 3. The dielectric permittivity is directly proportional to the polarization intensity of dielectric medium, where higher permittivity value gain in transformer oil produce better uniformity of the electric field in insulation medium. Hence, it is preferable to have a large value of permittivity for a good insulator. Referring to the temperature progression of the permittivity as shown in Fig. 3a, alloil samples are found to have a slight decrease in temperature except for disposed transformer oil without any filler. This occurrence was due to particles reduction, which resulted to a decrement in permittivity. Besides that, the polar molecules heat movements are aggravated when temperature increased, which hindered the orientation of the dipoles in electric field. Focusing on 25 °C temperature, the permittivity value for 0.005 g/L concentration obtain the highest value, while at 90 °C, 0.005 g/L, 0.01 g/L and 0.015 g/L concentrations obtain consistent permittivity value which is around 1.5. Nevertheless, the results of permittivity value for all concentrations were higher than disposed transformer oil.

Figure 3b depicts the dielectric dissipation factor (DF) for six concentrations of MWCNT nanofluids at various temperature. DF denotes the dielectric losses in insulating medium, which indicates total amount of contaminations in transformer oil. Nanofluids samples are observed to gain lower DF value with respect to ascending temperature compared with disposed MO. It is also found that disposed MO has a steeper slope compared to others, indicating the deterioration of insulation, contamination and degradation. As for MWCNT nanofluids, the DF is continuously below 0.02 from 25 to 120 °C temperature. It is ideal to provide DF as minimal as possible due to a low resistive current, which suggesting high resistive in insulation. Focusing on DF value at overload condition, it is observed that 0.005 g/L concentration obtain the lowest DF value while oil without MWCNT obtains the highest DF value. In general, DF is the ratio of energy loss to total energy transmitted through insulation medium. Based on IEC 60422^16^, if the DF value exceeds 0.04, catastrophic failure is reported.

Figure 3c demonstrated the comparison of resistivity for oil samples at ascending temperature. Resistivity is a fundamental property for quantifying how strongly insulation resists or conduct electric current. It is desirable to have resistivity of oil as high as possible. It is observed that the resistivity of the oil samples declines exponentially with rising temperature. Furthermore, nanofluid at 0.005 g/L achieves the highest resistivity along the temperature followed by 0.01 g/L and 0.015 g/L concentrations. Referring to 90 °C temperature (overload condition), 0.005 g/L MWCNT nanofluid also has the highest resistivity value reflecting the performance of BDV and LI breakdown voltage. High resistivity performance represents the low formation of free ions and conductive impurities. The level of ageing and the degree of contamination of the insulating oil also can be assessed by referring to resistivity.

### Raman structural properties

**Figure 4:**
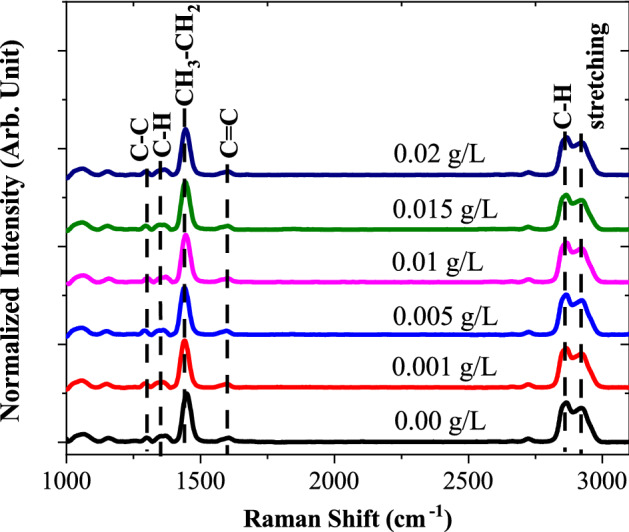
Raman spectroscopy analysis of oil samples at different concentrations.

Raman spectroscopy probes the chemical structure of material and provides information regarding chemical bonding identity, contamination and impurity. Figure 4 illustrated the Raman spectra of oil samples at various concentrations of MWCNT nanofluids from 1000 to 3100 cm^−1^ wavelength. It can be observed that there were a few low-frequency peaks existed below 1300 cm^−1^ region representing carbon–carbon C–C vibrations. The set of peaks at 1300 cm^−1^ and 1350 cm^−1^ corresponds to paraffin C–H twisting modes, while the large peak around 1440 cm^−1^ corresponds to CH_3_–CH_2_ bending mode. Furthermore, it is also observed that there is a peak existed around 1600 cm^−1^ which due to an aromatic C=C stretching mode. Focusing on higher wavelength, appear that two obvious peaks at 2860 cm^−1^ and 2920 cm^−1^ which attributed of carbon–hydrogen (C–H) stretching vibration which represent to diversity of CH, CH_2_ and CH_3_ group^17–19^. The carbon in the former is part of aliphatic CH symmetric stretching, whereas the carbon in the latter form part of aliphatic CH asymmetric stretching. In symmetric stretching, two or more bonds vibrate in and out together. In asymmetric stretching, some bonds are getting shorter at the same time as others are getting longer. It also can be observed that the C–H structure has higher frequency compared to C–C bonding structure. This is due to hydrogen which is much lighter compared to carbon atom^20^. However, based on overall observation of Raman shift, there was no additional peak existed in disposed transformer oil chemical structure after adding MWCNT which means that MWCNT molecules did not disrupt the structure behavior of transformer oil.

**Figure 5:**
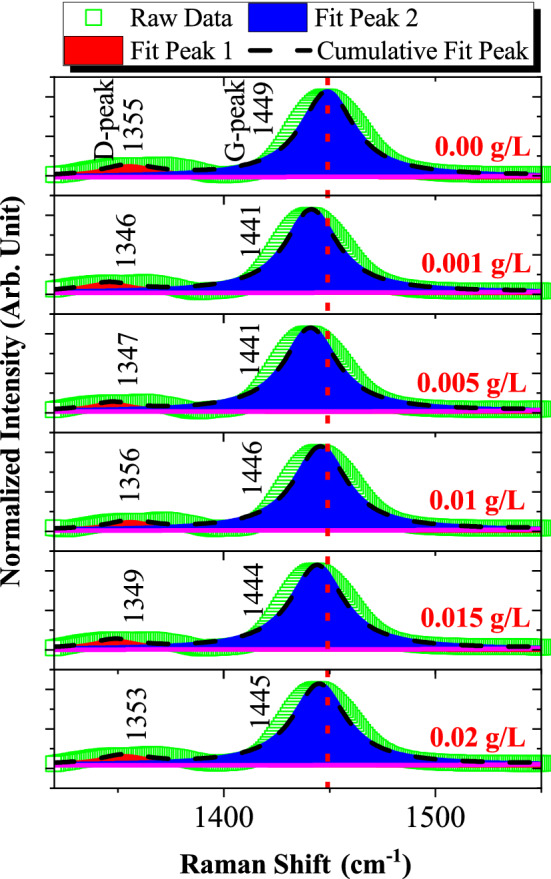
First-order region of Raman spectrum for various concentrations of MWCNT nanofluids.

Raman spectra of the oil samples were mainly characterized by first-order region (1400–1600 cm^−1^) and second-order region (2800–3000 cm^−1^). Within the first-order region, the disordered band originating in structural defects exists at around 1350 cm^−1^, which are referred as D-band, while most prominent band exists at around 1450 cm^−1^ known as the G-band (graphite). D-band arises because of the presence of defects or disorder; thus indicating a higher degree of disorder in this sample. The D and G peak positions were determined by a Lorentzian fit after baseline subtraction as shown in Fig. 5. It can be observed that, after dispersion of MWCNT in transformer oil, the low intensity peak existed around 1440 cm^−1^ shifting to a lower wavenumber. In Raman spectra, shifting of peaks towards lower wavenumber is related to longer chemical bond length of molecules. The shortening or lengthening the bond length is a result of changing inter-particle interactions.

**Figure 6:**
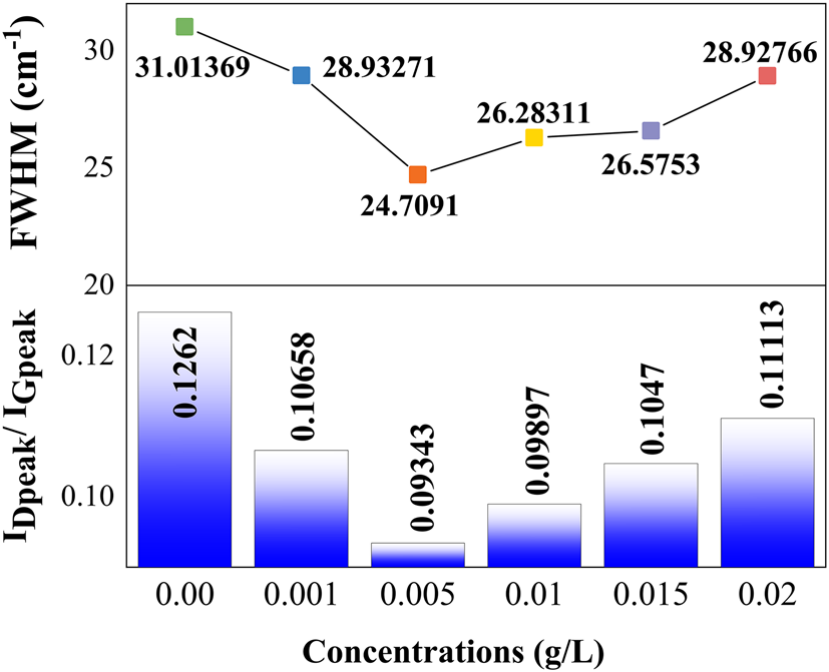
Intensity ratio of D-peak to G-peak and FWHM of oil samples.

Figure 6 depicts the calculation of intensity ratio of D-peak to G-peak (I_D_/I_G_), full width at half maximum (FWHM) and area under curves of D-peak at various concentrations of MWCNT nanofluids. Using the peak intensity of I_D_/I_G_ ratio, one can use characterized the level of disorder in structure. Based on Fig. 6, I_D_/I_G_ ratio of disposed transformer oil is 0.1262 which further validates the presence of high disordered in the samples compared to 0.005 g/L MWCNT nanofluid with I_D_/I_G_ ratio of 0.09343. Besides that, higher intensity of D-peak value confirms the existence of oxygen functional groups in the oil samples^21^. Other than observing the intensity data with respect to its wavenumber, the FWHM of D-peak is also calculated in this paper. FWHM is the width of a peak at half of its intensity which indicates the reflection of the structural distribution. The large FWHM of D-peak also indicates the severely reduced crystallinity and seems that the sonication process of MWCNT in disposed transformer oil brings no damage to the structure of C–H bonding^22^.

### Small angle X-ray scattering

**Figure 7:**
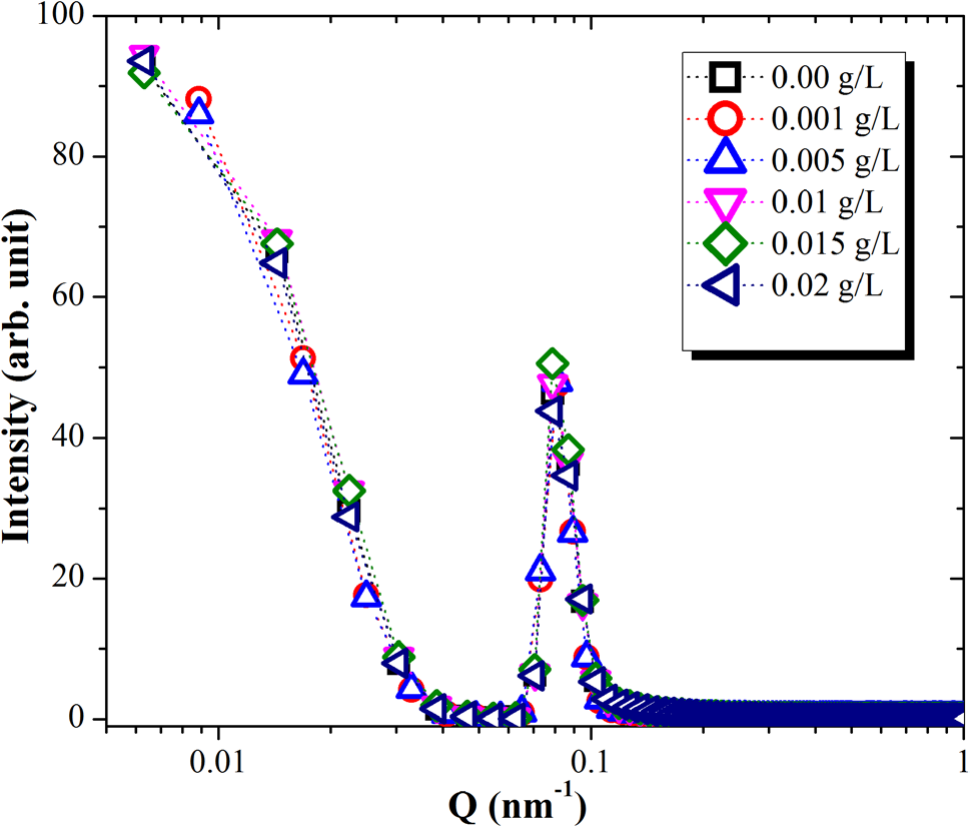
X-ray scattered intensity as a function of Q in double logarithmic scale for oil samples.

To better understand the structural effects of MWCNT on disposed MO, the crystallographic knowledge on solution structure with small angle X-ray scattering (SAXS) in room temperature condition is tested to characterize disassembly aggregates in samples. MWCNTs are ideal structural reinforcements for MO nanofluid due to their extraordinary mechanical properties such as flexibility, stiffness and extremely high Young’s modulus^23^. Essential steps need to been concern in involve dispersion of the MWCNT within the MO and processing of the nanofluid in order to transfer the outstanding properties of MWCNT in nanoscale to microscale of MO. Figure 7 shows the SAXS scattered intensity for an oil samples made of MWCNTs and MO. It seems that all weight concentrations of MWCNT nanofluids show perfect matches with the disposed MO without any filler SAXS scattering. This behavior is agree well with others group which their claim CNT dispersed in liquids using small angle neutrons and X-rays scattering study^24–26^. The scattering patterns exhibit a strong maximum around Q = 0.07 nm^−1^ related to the long spacing of the MO chemical structure matrix.

**Figure 8:**
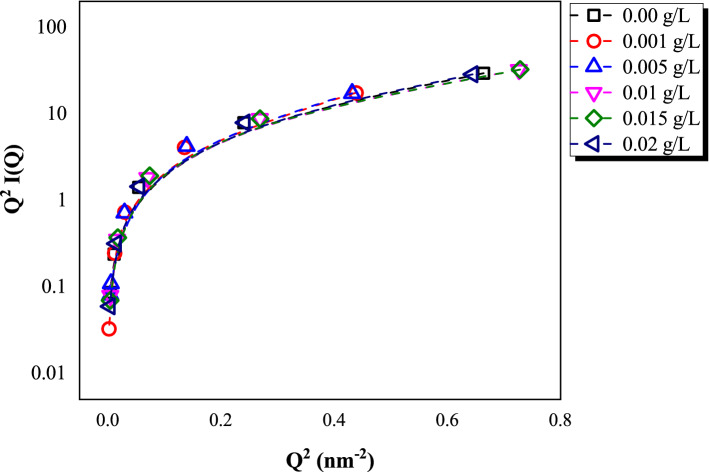
The Kratky plot of disposed mineral oil and various concentrations of MWCNT nanofluids ranging from 0.001 to 0.020 g/L.

Moreover, the SAXS data also provides several indicators such as to presence the chemical structure flexibility. For example, the Kratky plot as shown in Fig. 8; Q^2^(I(Q) as a function of Q is employed to quantitatively identify the folding states of samples. The scattering intensity will exhibit a “bell-shape” peak at low Q intensity region for the case of well-folded state. For this study, it is observed that all the oil samples show a rise in the curve where they are as unfolded states. This is due to the presence of electrostatic potential between particles, which is typically the result of Van der Waal forces in oil^27^. MWCNT consists of cylindrical shape which supposed to have stronger attraction between neighbor MWCNT and stronger tendency to form aggregation^28^. However, based on the Kratky plot, observed that introducing MWCNT in disposed MO molecules did not promote such aggregation. Perhaps, the MWCNT structure could bind to the particles of MO to form homogeneously dispersed nanofluids without requiring extra surface modification.

## Conclusion

Laboratory experiments on electrical and dielectric properties of multi-walled carbon nanotube (MWCNT) based disposed transformer oil at various concentrations (0.0 g/L to 0.02 g/L) were carried out in this paper. It was figure out that the suspension of 0.005 g/L MWCNT with disposed transformer oil manifested an enhancement of 212.58% in the AC breakdown performance followed by 168.87% enhancement for 0.01 g/L concentration compared to transformer oil without any nano-filler. As for negative lightning impulse performance, there was more than 12.29% improvement after adding some MWCNT nano-filler. Besides that, the dielectric permittivity, dissipation factor and resistivity also produced promising results that reflected the behavior of electrical performance. It can be concluded that the electrical and dielectric properties obtain the highest performance at a concentration of 0.005 g/L. This is due to the fact that at 0.005 g/L, the nanofluid tends to have better ability to manage thermal properties of oil and translate into greater convection heat transfer compared to other weight concentrations. For each nanofluid, there will be optimum value of weight concentration that will particularly affect the motion of particles in oil. In this paper, composition of material also has been recognized by utilizing Raman spectroscopy analysis and Small Angle X-ray Scattering in detailed. It was figure out that MWCNT nano-filler does not disrupt the chemical structure of disposed transformer oil at 0.001 g/L to 0.2 g/L concentration. Information provided from each testing and results considered together may form the development of new alternative transformer oil.

## Data Availability

The data that supports the findings of this study are available within the article.
